# Clinical scientist led healthcare in inherited cardiac conditions: a new frontier?

**DOI:** 10.1186/s44156-024-00049-w

**Published:** 2024-05-07

**Authors:** Stephen P Page, Gemma Bassindale

**Affiliations:** 1 Inherited Cardiac Conditions Service, Leeds Teaching Hospitals NHS Trust, Leeds, UK; 2 Association for Inherited Cardiac Conditions Council, Affiliated Society of the British Cardiac Society, London, UK

The last 50 years has seen an extraordinary transformation in our ability to diagnose, evaluate and manage individuals and families with inherited cardiac conditions (ICCs) [1]. The two most common conditions – hypertrophic cardiomyopathy and dilated cardiomyopathy can often be easily diagnosed and recognised by a wide range of health care professionals as diagnostic techniques become ever more powerful. The ability to detect subtle, or early disease in at risk family members however requires a high level of suspicion and specific knowledge and training. Clinical screening is therefore often performed in dedicated ICC centres.

To perform clinical screening effectively, clinicians need to be able to (1) accurately identify and reassure individuals that have normal tests (the large majority), and (2) accurately identify those with evidence of clinical disease expression (the small minority). For structural heart muscle disorders such as hypertrophic cardiomyopathy (HCM) and dilated cardiomyopathy (DCM), accurate identification of phenotype is based upon electrocardiography (ECG) and Echocardiography (Echo). Traditional clinical screening models involve a number of health care professionals, including individuals performing the ECG, trained sonographers performing Echo and a healthcare professional to make a clinical assessment to assimilate and interpret the results. Such models are therefore resource and time intensive.

The study by Draper et al. [2] describes a novel way of delivering clinical screening in a resource efficient and cost-effective way. They evaluated outcomes in a large Inherited Cardiac Conditions service in which clinical screening was delivered by a clinical scientist. They evaluated 200 first degree relatives of patients with the two most common forms of familial cardiomyopathy – HCM and DCM. The clinical scientist performed the entire clinical screening process including history taking, examination, ECG interpretation and performed and interpreted the Echo study. They identified that the significant majority (85%) had normal investigations and were able to discharge these individuals with the reassurance that they had no evidence of disease. In the remaining 15% they identified an abnormality requiring further evaluation, performed by specialists within the service, and ultimately made a clinical diagnosis in 10% of the overall cohort. Critically, given the age-related penetrance of these conditions, they were able to provide specific surveillance screening recommendations for ongoing assessment.

The key finding from this study is that clinical screening can safely and effectively be provided by a single trained clinical scientist in a single assessment, freeing up time and resources in specialist nurse or cardiologist delivered clinics. Having a single individual perform the entire screening process creates capacity for other clinicians within the service, to focus on patients with evidence of clinical disease, rather than the assessment of large numbers of relatives who are clinically unaffected. Further efficiencies were made by offering self-referral to at-risk relatives, obviating the need for formal referrals processes, and creating additional resource savings in primary care.

As the second largest workforce within the NHS responsible for delivering diagnostic investigations, clinical scientists are ideally placed to provide such clinical assessments. Traditional training pathways for cardiac clinical scientists ensure high level competency in ECG interpretation and in performing and interpreting echo alongside leadership and clinical assessment skills. The Modernising Scientific Careers pathway identified the potential of this group to diversify the way we meet the increasing demand within cardiology [3]. This has led to the development of structured training pathways towards consultant clinical scientist level meaning there is the potential for clinical scientists to provide consultant level care for clinically complex patient cohorts in future.

In a time when clinical resources are increasingly stretched in many healthcare systems, being able to streamline assessments by appropriately trained clinical staff is highly attractive and is likely to become an established alternative model of healthcare. The key for policy makers is recognising that the critical step is ensuring that healthcare professionals have the appropriate skills rather than focussing on their specific roles.

It will be fascinating to see how these new models of healthcare evolve over the coming years.

## Data Availability

Not relevant.
